# Object Learning Improves Feature Extraction but Does Not Improve Feature Selection

**DOI:** 10.1371/journal.pone.0051325

**Published:** 2012-12-12

**Authors:** Linus Holm, Stephen Engel, Paul Schrater

**Affiliations:** 1 Department of Psychology, University of Umeå, Umeå, Sweden; 2 Department of Psychology, University of Minnesota, Minneapolis, Minnesota, United States of America; 3 Department of Computer Science, University of Minnesota, Minneapolis, Minnesota, United States of America; Nothwestern University, United States of America

## Abstract

A single glance at your crowded desk is enough to locate your favorite cup. But finding an unfamiliar object requires more effort. This superiority in recognition performance for learned objects has at least two possible sources. For familiar objects observers might: 1) select more informative image locations upon which to fixate their eyes, or 2) extract more information from a given eye fixation. To test these possibilities, we had observers localize fragmented objects embedded in dense displays of random contour fragments. Eight participants searched for objects in 600 images while their eye movements were recorded in three daily sessions. Performance improved as subjects trained with the objects: The number of fixations required to find an object decreased by 64% across the 3 sessions. An ideal observer model that included measures of fragment confusability was used to calculate the information available from a single fixation. Comparing human performance to the model suggested that across sessions information extraction at each eye fixation increased markedly, by an amount roughly equal to the extra information that would be extracted following a 100% increase in functional field of view. Selection of fixation locations, on the other hand, did not improve with practice.

## Introduction

Object recognition is a hard problem. In natural images, objects can appear in unknown angles, can be partly occluded, and can be embedded in complex visual environments. Complicating the problem further, many objects are visually similar to one another. Yet despite these difficulties, humans recognize familiar objects easily and effortlessly, even against relatively complex backgrounds and their ability to do so improves from infancy well into adulthood [1]. Moreover, at any stage of life, increasing familiarity in a visual environment improves object recognition in that domain [2]. This raises the question: how does object familiarity make object recognition easier?

One possible strategy to aid recognition for familiar objects is for the observer to select the most informative parts of objects for further processing by focusing the eyes upon them. Specifically, subjects might fixate upon the parts of the image that contain the most diagnostic information for object recognition. This kind of selection strategy is common in many visuomotor domains such as grasping [3], [4] in which ideal finger locations are typically fixated while reaching for the object. Furthermore, visual search studies suggest that humans can learn to move their eyes to the most informative location, in this case to regions where a target might plausibly be found [5], [6], [7]. For instance, Torralba and colleagues [7] found that human observers constrained fixations to likely target locations when looking for familiar objects in everyday scenes. Even more compelling support for efficient selection was presented by Najemnik and Geisler [5] who showed that human observers made close to ideal eye movements in search for a simple gabor patch embedded in 1/f noise.

It is yet an open question whether object familiarity leads to optimization of information selection in object recognition. Findings that humans can recognize objects within 150 ms [8], [9], and in the near absence of attention [10] suggest that there is little need for selecting informative regions. Instead, familiarity might operate by combining more features to efficiently discern the object within a single glance. A correct integration of a large number of features should produce high object sensitivity, without the additional need of - or influence over - selection. That is, object familiarity might not guide attention or eye movements across the visual scene. To date there have been no explicit tests of whether familiarity amounts to improved information selection - or to the amount of information acquired in a single view. To determine the influence of object familiarity requires an overt indicator of selection and a method to quantify the information contained in a selected image region.

Measurements of eye movements open a window onto information selection during object recognition. Because objects in the natural world mostly appear against complex backgrounds, often in the visual periphery, humans need to redirect their eyes in order to acquire sufficient information to recognize objects. Peripheral vision is usually insufficient for object recognition because acuity drops off rapidly towards the periphery (at five degrees eccentricity, visual acuity is reduced by 50% [11].) Crowding of objects in the periphery presents an additional problem. For example, an isolated letter presented in the peripheral field might be easy to recognize, but when presented together with two other flanking letters, it becomes impossible [12]. Similarly, when objects are embedded in natural scenes, they become more difficult to recognize [2], than when they are presented against a plain background. Thus, the location of the eyes, which changes position about three times a second [13], should indicate which parts of the image are selected and used in recognition.

In this study, we examined whether familiarity with objects aids recognition by guiding information selection, as measured by the locations of the eyes. If this hypothesis is true, then eye fixations should move to more informative parts of an image as a subject gains experience recognizing an object. As an alternative, we consider the possibility that familiarity does not aid information selection, but instead improves the amount of information extracted at a given location. We test between these two accounts by using a formal model to evaluate the information available at fixation locations selected by human observers as they practice and improve in an object recognition task. Subjects searched for objects in noisy displays, a task which required them to make eye movements. Importantly, the size and composition of the objects relative to the background made certain parts of the object formally more diagnostic of its presence. If familiarity affects information selection, then more diagnostic object parts should be fixated more frequently as object search experience increases.

## Results

### Stimulus Generation and Task

**Figure 1:**
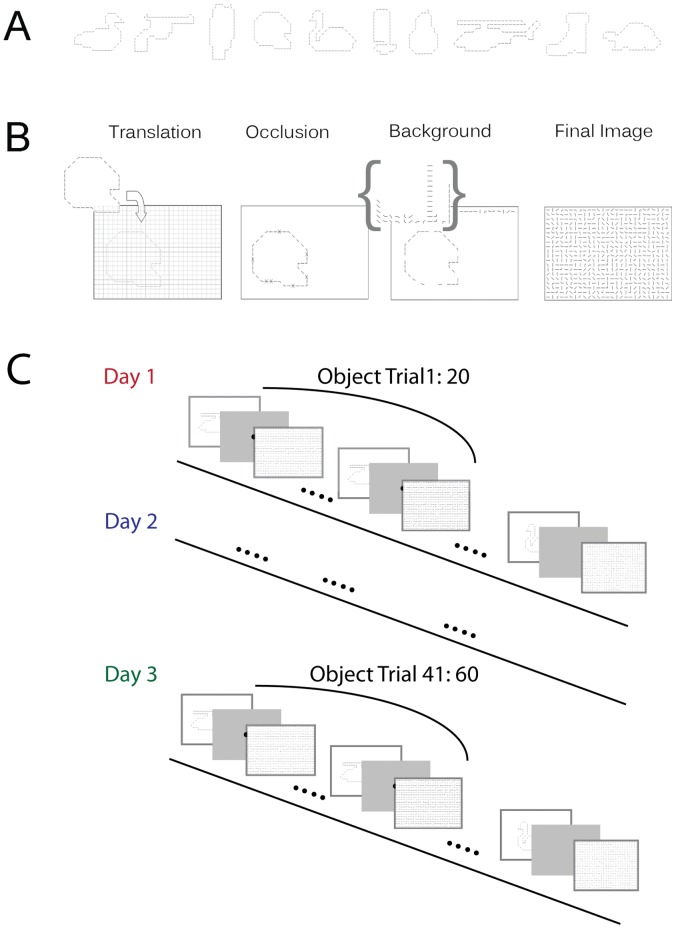
Stimulus and experimental design. (**A**) The ten object shapes used in the experiment. Objects were fragmented as shown. (**B**) Objects were randomly positioned within a 27×20 grid. Each object fragment was removed with p = .25. Finally all empty cells were filled with randomly selected fragments from the same object. Sixty images were generated in this way for each object. (**C**) Experimental design. Participants searched cued objects blocked in 20 trials per object. The cued object was first presented for 3s, followed by a central fixation dot. Participants fixated the dot and pressed a button to initiate the search image. Participants were told to press a button as soon as they found the object in each image, and then to continue to look at the object until the image disappeared, which occurred after 3 s. Object presentation order was counterbalanced across participants.

We used an object localization paradigm to test whether practice with a target object allowed observers to move their eyes to more informative image regions, or whether it increased their ability to extract information from a given region. The stimuli and task were designed to capture properties of natural complex scenes, while simultaneously being unfamiliar enough to allow for large amounts of learning. To make the search task difficult, yet tractable for an ideal observer, we fragmented objects into contours and embedded them in a background of similar contour fragments (Figure 1). The full image formation model is described in the methods section. The objects were randomly positioned on a grid that contained a randomly drawn fragment in each cell. Objects covered on average 23% of the image, or about 11×11 deg^2^ of the observers’ field of view (see Methods , and object templates in Figure 1A). As a control, all images were evaluated using the saliency toolbox [14] to test if the most salient image area also indicated object location. The saliency algorithm performed at chance, suggesting that basic visual attributes were insufficient to indicate object location in the images. To locate the object then required integrating multiple object features. The subjects’ task was to search for a cued object in the grid and respond by fixating upon it (trial procedure displayed in Figure 1C).

**Figure 2:**
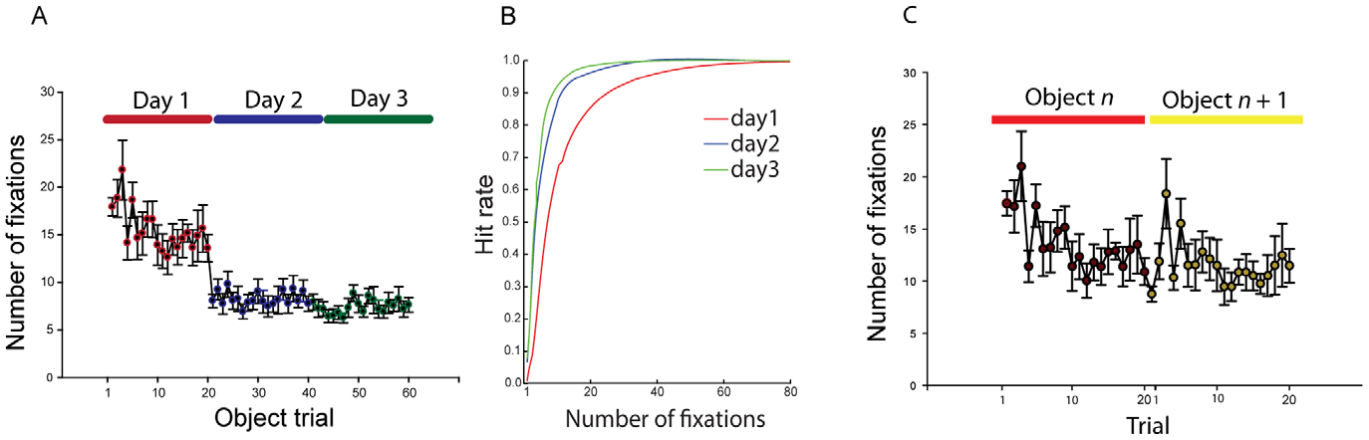
Learning results. (**A**) shows numbers of fixations to detection as a function trial number. (**B**) shows hit rate as a function of number of fixations on days 1, 2 and three. All error bars are SEM over participants. (**C**) shows number of fixations as a function of trial for adjacent object blocks on day 1.

### Empirical Results: Object Search

To test how familiarity affects information extraction we recorded the eye movements of eight participants as they searched for fragmented objects (one at a time) in 600 images across three sessions. First, we established that participants generally improved in their search performance by testing search speed and accuracy across the three daily sessions (see Figure 2). Mean number of fixations until response went from 13.1, to 5.5 and 4.7 days 1 through 3. The decrease was statistically reliable, F(2,7) = 27.8, p<.0001. The corresponding response time trial averages were 4.2 s, 1.8 s and 1.5 s for days 1 through 3. The response time decrease was also statistically reliable, *F*(2,7) = 32.8, *p*<.0001. This did not reflect a speed accuracy trade-off, as hit rate at the end of the trial (determined by fixating on the object at button press) remained almost constant; at approximately *M* = .92 on each session. As seen in Figure 2B, hit rate per fixation improved across sessions. The location of the final fixation (i.e., at recognition) also remained relatively constant. Distance from the object center was on average 4.66, 4.54 and 4.61 degrees days 1 through 3 (*F* <1). Saccade amplitude was also relatively unaffected by learning, with means of 5.1, 5.1 and 5.5 on days 1 through 3 (*F*(2,14) = 2.33, *p*>.13).

**Figure 3:**
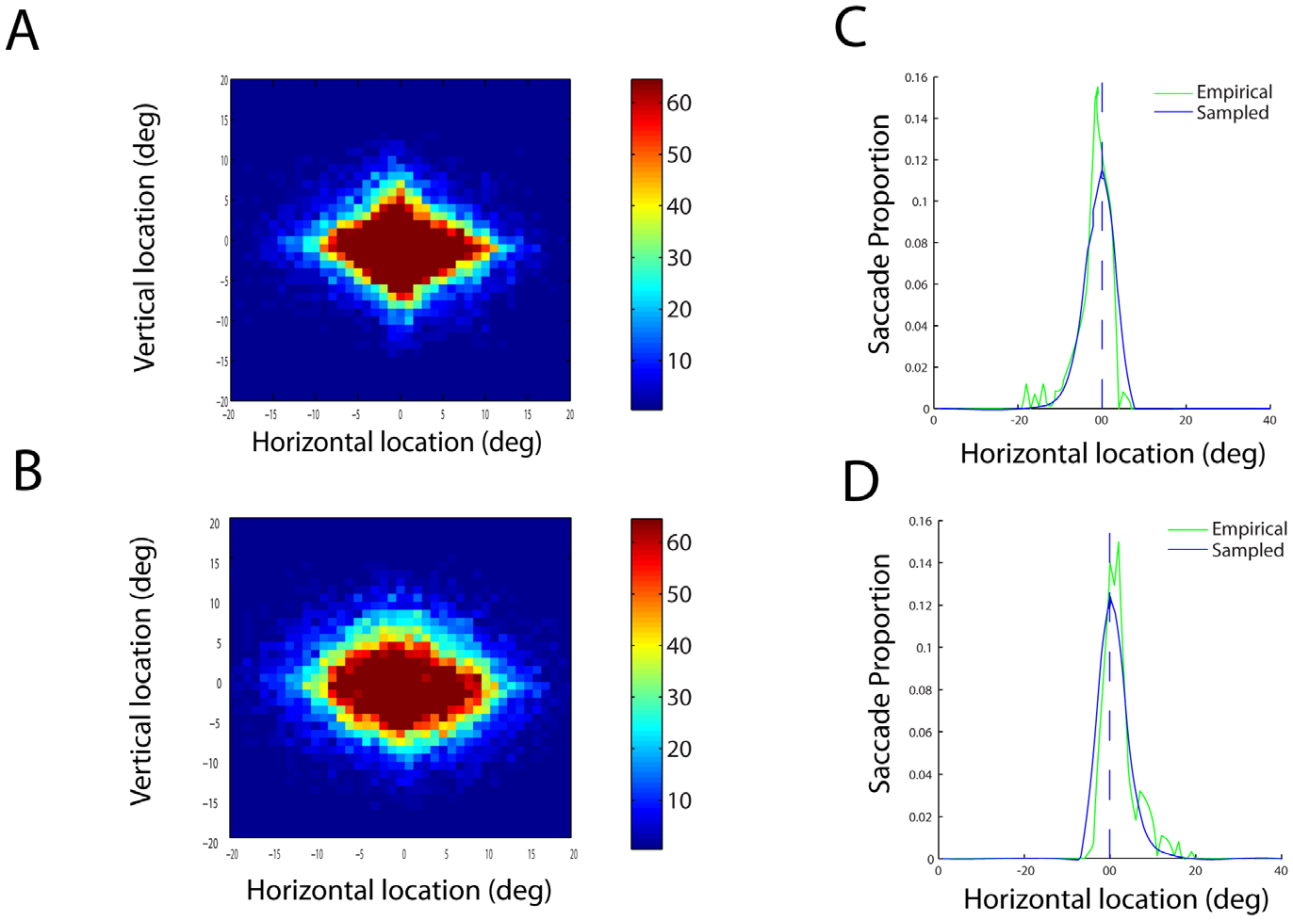
Fixation distributions. (**A**) Empirical fixation distribution (data based on all recorded eye movements) for current fixation subtracted by last fixation (P(f_i_|f_i-1_) and (**B**) the empirical distribution for current fixation subtracted by the next to last fixation (P(f_i_|f_i-2_). Hot maps reflect number of fixations. Right graphs show distributions of horizontal saccade vectors, given that the previous saccade was directed to (**C**) the right or (**D**) left.

**Figure 4:**
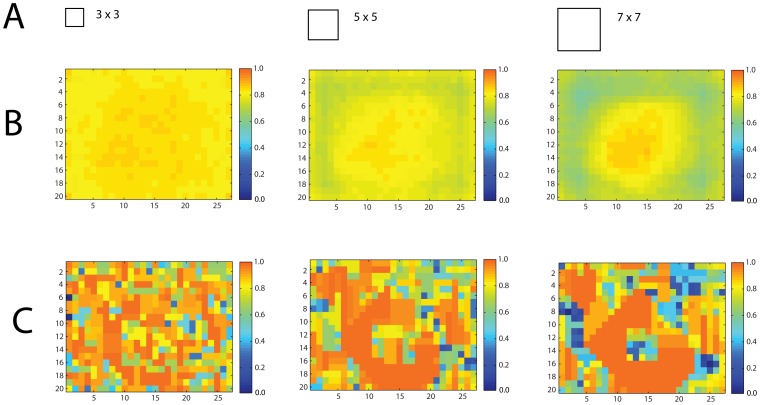
Object likelihood computations based on three different fields of view. (**A**) is field of view from a single fixation relative to the image (**B**) shows stimuli average (600 stimulus images) object likelihood distribution given the field of view (**C**) is corresponding likelihood distribution for a single stimulus image (same stimulus image as in figure 1B). Hot maps indicate object likelihood, image axes are coordinates in degrees.

Much of the observed learning appeared to be object specific. Trials were arranged in blocks of 20, with each block using a single object and each object appearing in a single block per day. The number of fixations within a block decreased steadily from trial to trial, but returned to close to initial performance as participants started on a new block (see Figure 2C). It then gradually decreased for the new object. This pattern is typical of object specific learning.

The decrease in search time might indicate that participants just changed their response bias across sessions, and not necessarily that they became more efficient in locating the object. However, number of fixations until the object was first fixated in the trial decreased with practice, from a mean of 1.78 on day one,to 1.53 and 1.56 fixations on days two and three, respectively (F(2,14) = 29.6, MSE = .005, p<.0001). This pattern of results does not appear to be consistent with a simple change in decision bias because looking at the object should always be more informative in the localization task, regardless of decision bias.

To conclusively determine that the training improved recognition performance, a simplified control experiment involving eight new participants was conducted. In the control experiment, the participants first made two alternative sequential forced choice (2AFC) detection judgments with similarly generated images. After the initial detection test, participants made 20 object localization trials identical to those used in the main experiment. Subsequently, participants were again tested on the object detection test with new image draws from the generative model. The results showed a detection improvement present in every participant with a detection hit rate going from *M* = .66 to *M* = .76 for pre and post search detection test, respectively, which reflects a change in sensitivity rather than bias because the task was 2AFC. The difference between pre and post-test was highly significant at *t*(7) = -5. 68, *p*<.001. Taken together then, the results suggest that participants genuinely improved in object recognition.

**Figure 5:**
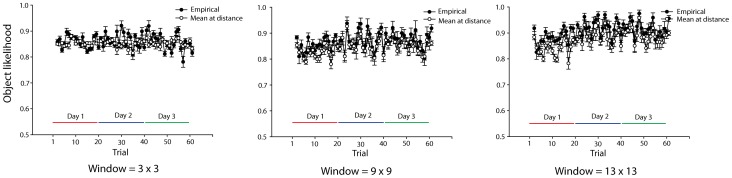
Object information per fixation as a function of practice calculated for three fields of view. Separate lines for the empirical fixations (black) and the average information of image locations at the same distance from the image center (white). Error bars are SEM across subjects.

**Figure 6:**
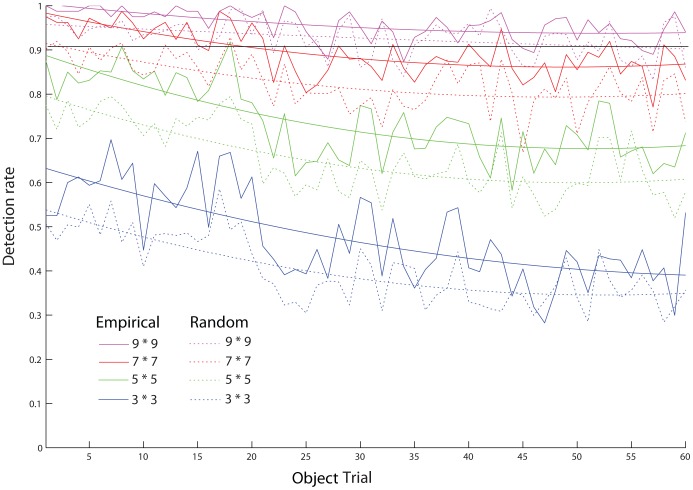
Detection performance as a function of trial for the ideal observer. Solid lines indicate performance given the empirical fixations. Dashed lines are based on random walk fixation sampling (100 samples per trial, subject and object) using the same number of fixations as the subjects in the corresponding trial. Smoothed curves are fitted 2^nd^ degree polynomials to sampled and empirical data, respectively. Black horizontal bar reflects detection rate at. 9.

To determine the *nature* of this performance improvement, we now turn to an analysis of *how* fixations were distributed as a function of practice. Specifically, did participants need less time and fewer fixations because they selected more informative image locations with practice or because they learned to integrate more visual information in each fixation?

### Empirical Distribution of Fixation Locations

One way to test formally for improvement of fixation locations is to examine their temporal independence. If observers select fixations intelligently, based on the potential for new diagnostic information, then they should use information from previous fixations to guide their next fixation location. For example, in the simplest case, observers should avoid resampling image locations that they have already visited. Figure 3A illustrates the distribution of fixation locations with respect to the last or next to last (Figure 3B) fixation. The distribution is roughly Gaussian, which suggests that there is little avoidance of returning to previously sampled image locations; had there been such, Figure 3B would have an annulus-like distribution. Rather, the distribution is close to what would be expected under random selection of image locations.

A closer examination of the data suggests that there was some spatial constraint affecting the fixation distribution. For instance, a saccade to the left increased the probability of the next saccade being to the right. However, that property would also be expected under the simple assumption that the observer constrained his or her fixations to stay within the image. To test whether this can account for the data, we implemented a random walk model starting out at the same central image location as humans. The model’s saccades were independent draws from the overall empirical distribution of saccade vectors, with the constraint that those that would have ended outside the display window were discarded and replaced by another random draw. As seen in figure 3C–D, the random walk sampling procedure produced a similar spatial dependency to the empirical distribution.

**Figure 7:**
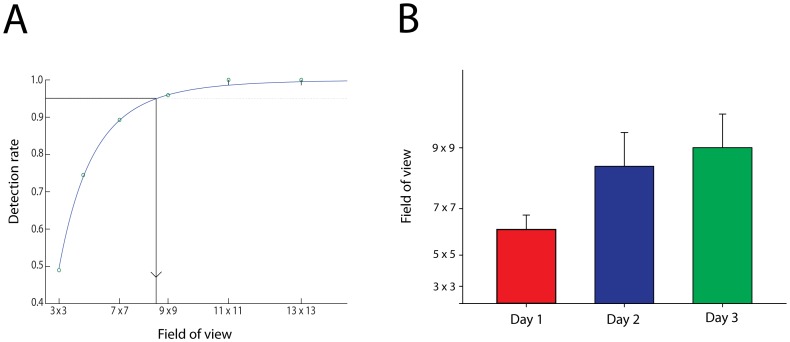
Functional field of view. (**A**) Logistic fit to ideal observer performance calculated for six different field of view sizes. The ideal observer performance was calculated using each subject’s empirical fixation distribution for a particular day. Inset dashed horizontal bar indicates the subject’s performance that day. Intersection between logistic fit and subject detection rate is the estimate of the subject’s field of view that day. (**B**) Estimated fields of view across the three daily sessions, error bars are SEM across subjects.

**Figure 8:**
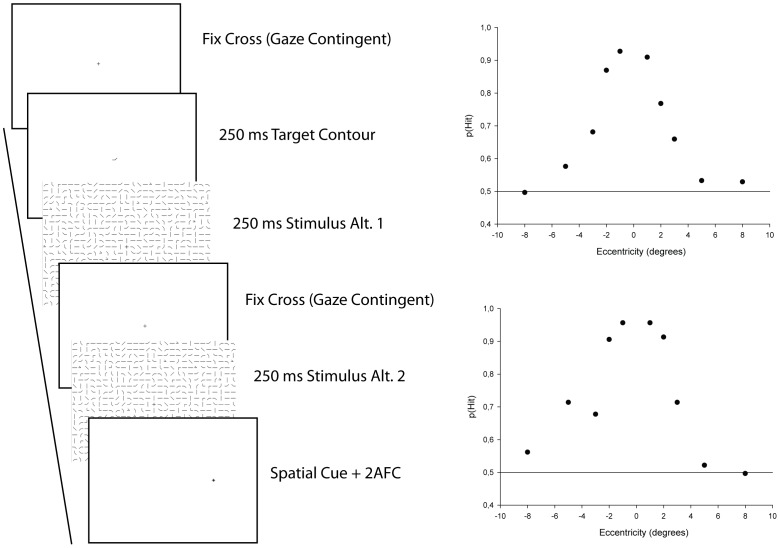
Feature visibility. Left panel shows psychophysics procedure to establish fragment visibility. Right panel shows average feature hit rate as a function of horizontal (top graph) and vertical (lower graph) eccentricity. The 0.5 horizontal bar indicates chance level.

### Ideal Observer Analysis of Fixation Locations

To test even more strongly if selection improved with practice, we implemented an ideal observer that quantified the amount of information at each fixation that could be used for the object identification task (see Methods). Note that the observer used here is not a true ideal, but is constrained by a field of view parameter that determines the radius surrounding fixation within which information is extracted. For human observers, we do not know the size of the functional field of view. However, modeling the field of view size in the ideal observer allows us to test selection efficiency under any field of view in human observers.

To estimate the informativeness in any part of the image, the ideal observer computes the likelihood ratio between the probability that the observed features within its field of view are produced by each possible object subset and the probability that the features are produced by the background. An object subset is any set of spatially preserved object features that fit within the ideal observer’s field of view. For a given field of view and image location, the model selects the object subset that receives the highest likelihood as its best guess for where the object is located in the image. The selected likelihood will thus vary across image locations. For instance, in the region of the image where the object is actually located, likelihoods will typically be higher, because there is more evidence for the objects presence there.

**Figure 9:**
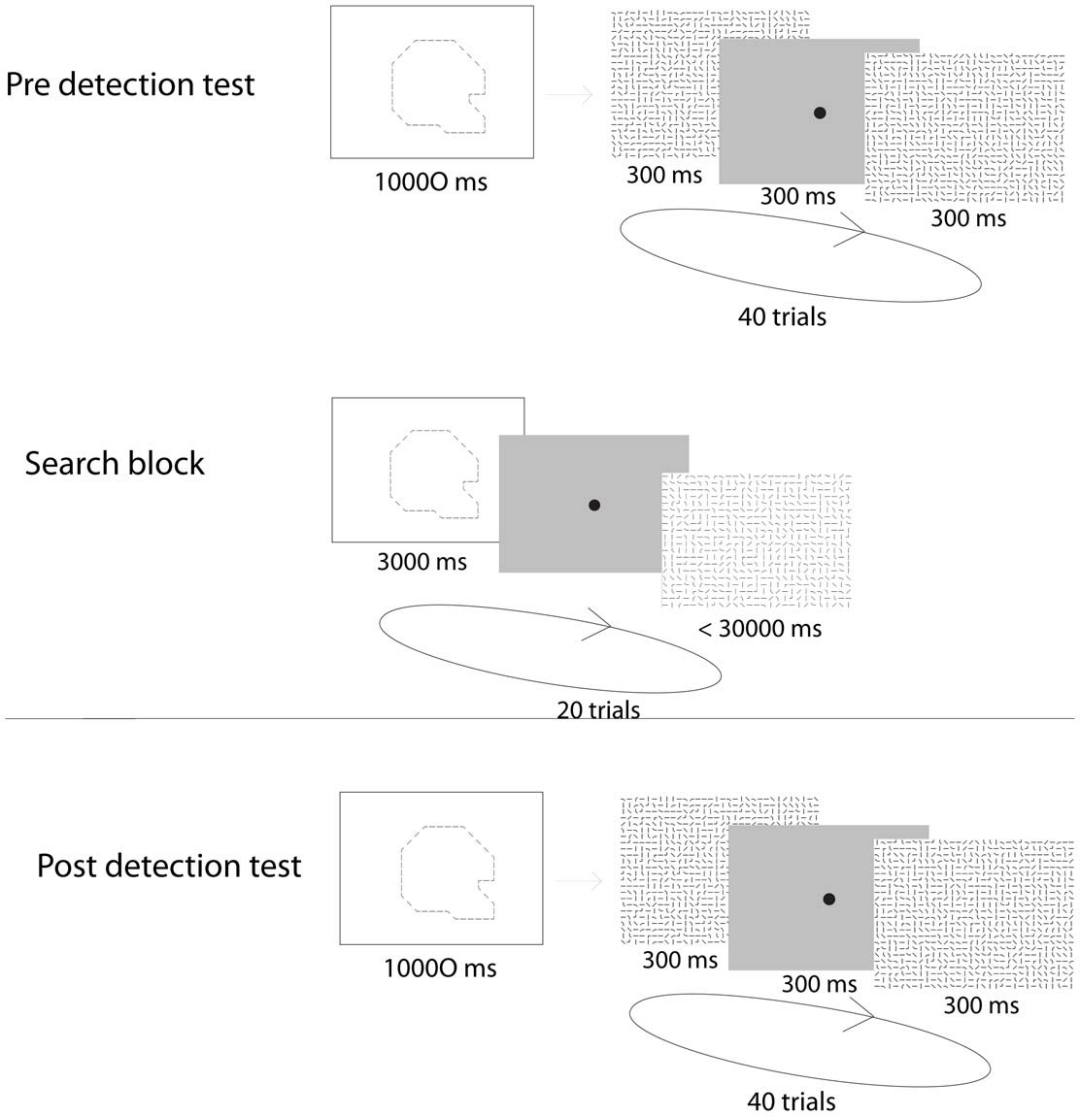
Control Experiment Object detection. Experimental design of control experiment. Image corresponds to one test block. Each participant was tested in three such blocks.

**Figure 10:**
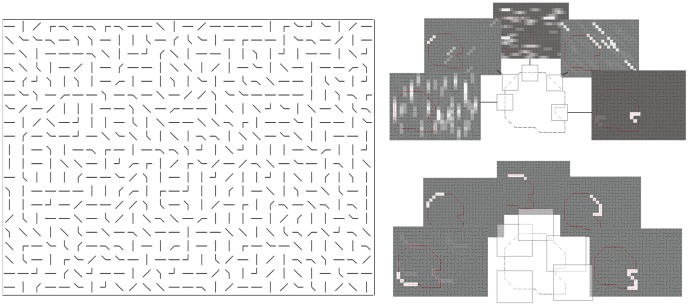
Fragment informativeness. Left panel shows a stimulus image produced with the helmet object. Right panel shows the likelihood ratio values for five different object subsets from a 3×3 window as intensity values (upper image) and subsets from a 7×7 window (lower image).

Figure 4 shows how the field of view parameter affects model performance. Specifically, larger fields of view receive on average more object information from the center of the image (see 4B). Also notice that for an individual image, the object information is clustered around the object’s outline, and wider fields of view produce a higher tolerance for fixation locations in the object’s vicinity (4C). Also, the small window will typically produce fairly high likelihoods all over the image (see first column in figure 4). This also means that the model with a small field of view will tend to produce false positives. Instead, a larger field of view will produce more accurate discriminations as well as a more accurate likelihood distribution at the individual image level (c.f., row 4C between the 3^2^ and 7^2^ window images).

We used the model to test whether human fixation locations contained more information than randomly selected image locations. Because the objects had to fit completely within the display, locations at the center of the screen contained more information, on average, than those towards the edge of the screen (see figure 4B). To take that property into account, we compared the information at each human fixation to the average information of image locations at the same distance from the image center (Figure 5) thus controlling for the expected higher information values at central regions of the image.

As seen in figure 5, there were no obvious differences between information at human fixations and the average information content at the same distance from the image center. We quantified this observation by aggregating trials into daily sessions and tested the difference in a set of 2(empirical vs. random)×3(day) repeated measures ANOVAS, one for each of eight different fields of view (from 3^2^ to 17^2^ by twos). Doing these analyses across a wide set of fields of view is motivated by the fact that we do not know the functional field of the human observer. However, if some field of view produces significantly higher information than chance it might indicate that it corresponds to the human observer’s field of view and that the human selected efficiently. Instead, if none of the tested fields of view showed significantly more information at actual than at random fixation locations, then it would support the notion that participants selected randomly. Furthermore, testing information at fixation as a function of session allows us to test whether human improved selection with practice.

The results of this analysis showed that the empirical (i.e., participants’) fixation locations contained slightly more object information than random locations, but that this difference did not increase with practice, and so cannot account for the observed learning effects. Participants’ fixations were significantly higher in information content than the average at the same distance from image center regardless of field of view used (all p< = 0.02). While significantly higher, the difference was very small, varying between.004 and.035 in likelihood difference across the eight field of view sizes. There was a small increase of information with practice for fields of view between 7^2^ to 17^2^ degrees (p< = 0.03) but more importantly, there was no significant *interaction* between random and empirical fixations, for all fields of view < = 9^2^, (all *F* <1.4. Had there been any improved selection, the information content in the empirical fixations should increase with practice, but the information content in the random should remain virtually stable. Additionally, for large fields of view (i.e., > = 11^2^) there was an interaction showing *less* difference between empirical and random fixations across days, p< = 0.05. In other words, if participants integrated feature information from fairly large regions of the image, practice made their selection *more* random.

As a final test of whether improvement in fixation locations could account for observers learning on the task, we extended the ideal observer, allowing it to integrate information across fixations and complete the object identification task. This was accomplished by taking the fixation with the maximum object likelihood and testing if its most likely object fragment was on the object (also, see equation 6 in the methods section). That is, for each empirical trial, the ideal observer determined which object subset location received the highest likelihood across all fixations and object subsets, using a fixed window size (out of eight possible, see figure 6). If the most likely fragment and location were on the object in that image, the result was scored as a hit (see equation 6, methods section), otherwise it was considered a miss. Note that the model has no learning, that is, this analysis assumes unchanging information extraction at a given location across trials.

This ideal observer analysis strongly suggests that subjects’ improvement in the task was not due to selection of more informative locations. Recall that subjects’ ultimate performance was constant across days, but that this was achieved with fewer and fewer fixations. If this pattern was accomplished by subjects selecting more informative locations, then the total amount of information extracted by the ideal observer should remain constant, despite the decrease in the number of fixations. As seen in Figure 6, however, the total amount of information extracted, as indexed by the ideal observer object recognition performance, actually decreased across trials. As subjects practiced the task, they required fewer fixations per trial, while not changing the informativeness of each fixation location, which resulted in less total information extraction from the unchanging ideal observer. Effects of practice on the task must be due to some other mechanism than improved selection of fixation locations.

This analysis also rules out the possibility that simple strategy changes can account for the observed learning, for example “staying in the center of the image.” If such strategic changes were sufficient, then the model should have found increased information at each fixation location large enough to keep performance constant. Instead, ideal performance declined when subjects’ actual fixation patterns were used with unchanging information extraction.

To test how different from random selection human observers were, we again simulated a random sequence of fixations, drawn from the empirical distribution of saccade vectors. Performance of the ideal was worse using these than using the actual empirical fixation sequences, suggesting that subjects were in fact somewhat strategic in their fixation selection. However, the difference in performance was constant over trials, which indicates that task learning was not due to improved selection of fixation locations (see Figure 6).

### Field of View as a Function of Learning

The above analyses rule out changes in selection of fixation location, and so suggest that subjects learning must have resulted from an increase in the amount of information they could extract at each fixation. To quantify the size of this increase, we computed ideal observer performance using the empirical sequences of fixation locations for a range of fields of view. Because each field of view size and set of fixations will produce a certain hit rate (see above and the ideal observer model in the methods section), we get a range of hit rates as we vary the modeled field of view. Specifically, the larger the field of view for any given set of fixations, the higher the hit rate. The relationship between hit rate and field of view produces a relationship that is well captures by a logistic function (see Figure 7A).

We computed the functional relationship between each participant’s fixations for each of several fields of view and thus established a participant and session specific logistic function. We then estimated the participant’s field of view by reading out the field of view value corresponding to the participant’s average hit rate on that session (see Figure 7A). Average window sizes thus estimated were 6.4^2^, 8.6^2^ and 9.3^2^ degrees for days 1, 2 and 3, respectively (see figure 7 B). The increase in window size was statistically reliable at F(2,7) = 5.52, p<.015. This estimate corresponds to a more than two-fold increase in the visual field area across session days. This result should not necessarily be taken as a process model of subjects’ performance; we do not know if subjects were actually expanding their functional field of view with practice. But the analysis does reveal that practice enabled subjects to extract additional information from a given fixation location of an amount equivalent to a substantial increase in functional field of view.

## Discussion

This study tested how familiarity with objects affects visual information acquisition by investigating how information is selected in an object localization task. Two possibilities were identified; object knowledge might improve selection of fixations and hence the quality of information received, or knowledge might increase the amount of information acquired in a fixation. The results strongly suggest that human observers receive more information in each fixation with practice, equivalent to a twofold increase in field of view, but do not choose fixation locations more efficiently. Anecdotally, several subjects claimed they relied on details while searching day 1, but learned to “match” the entire object when searching days 2 to 3. Similarly, participants in the control detection experiment mentioned pop-out like experiences of the objects in the post search detection test, but not in the pre-test.

The ideal observer model assumes a template-like representation of the object and we expressed the information increase in terms of a square field of view. Neither of these descriptions needs to be valid reflections of human object recognition given the present findings. For instance, humans might hold a much fuzzier representation of the object and the functional field of view could have been constant, but its content processed more efficiently with practice. Also, the all-or-none field of view model employed here might be further improved with a model that takes human gradual acuity fall-off to periphery into consideration. Regardless, the results support the idea that human observers learn to use more joint visual statistics as they improve in object recognition. This should be true for a range of statistics beyond those tested in this study, such as spatial frequency, context regularities, color, motion etc.

It might seem surprising that fixation selection was not better informed (see e.g., [5], [6], [7]). For instance, Najemnik and Geisler [5] found that human observer’s performance in a search task for gabor patches in 1/f noise was consistent with ideal selection of fixations. The task in the present study employed much more complex stimuli in that integration of higher order visual statistics was required to distinguish object from background and that the object was significantly larger, spanning roughly 11×11degrees of the visual field. This additional complexity introduced an object recognition difficulty not present in [5]. For instance, immediate object fixation was typically not sufficient to produce localization. Furthermore, there was no spatial prior the participants could have utilized across trials, as each object was uniform randomly translated in the image, hence removing the spatial cues present in natural scenes [7].

Droll and colleagues [15] found that participants learned the cue validity of neighboring distracters in a simple search target with practice. It is possible that we would have received similar patterns of results, had the targets been sufficiently simple. Instead, the complexity and continuity constraints imposed by the object contours in the present study might have afforded a more efficient learning by supporting wider information integration. This in turn reduces the value of accurate fixation selection.

We acknowledge that subjects were not entirely random in selection, as the empirical fixations were consistently associated with a higher detection rate than a random walk of similar window size would credit (see figure 6). It is difficult to judge what selection principles account for the difference at this stage. Regardless, this slight benefit did not improve with practice, and therefore cannot account for learning. Given more practice, better selection might yet have developed: Future studies could investigate if and when such learning takes place.

Some earlier search studies also reported random or virtually random fixation selection [16], [17], consistent with our results. We suggest the seeming discrepancy between [5], [6], [7], [15] and [16], [17] might reside in the utility of planning. First, if there is no or weak information integration across fixations, resampling old locations by refixating them might provide new insights to the object location in the present study. Therefore, avoiding old locations is not necessarily beneficial to the observer. Second, computing efficient saccades under uncertainty might take time. In the trade-off between more fixations versus higher quality fixations, the ideal computational solution for the observer might be to make more but random saccades because image processing is efficient but planning is not.

## Methods

### Ethics Statement

All experiments involving human participants were approved by the institutional review boards (IRB) at the University of Minnesota and/or the University of Umeå. All participants gave written informed consent in compliance with the Helsinki declaration.

### Object Search Procedure and Materials

Eight university of Minnesota students (age M = 19.25, four male) participated for course credit.

Ten different objects were used as search targets (see Figure 1A) and were randomly positioned in images that covered 27×20 degrees of participant’s visual field, corresponding to 27×20 image cells (see Figure 1B). The target objects varied in size from requiring a 15×8 cells patch to a 16×16 cells patch to cover the object. The objects templates enclosed an area of between 109 image cells (pear) to 155 cells (boot) corresponding to between 20% and 29% of the image. The images used in the experiments contained 27*20 cells. The average area covered by the objects was 127 cells (see Figure 1A). The average eccentricity from the image center to object center was 3.54 degrees, *SD* = 1.28. Each 1×1 degree cell contained a line segment from the background or the object contour. The segment was fitted within the boundary of the cell so that the segment ended 3 (cardinal orientation endpoints) or 6 (oblique orientation endpoints) pixels from the boundary thus forcing fragmentation of the image content. Images were presented on an LCD monitor (22″ dell-2209 wa) at 810×600 pixel resolution. Eye movements were recorded during all sessions (Eyelink 2, 500 Hz).

Participants searched for the ten cued objects presented in blocks of 20 images for a single object (see trial procedure in Figure 1C), giving 200 trials per day in each of three days. Before each search trial, the intact search target object (i.e., the cue) was presented in the center of the screen for 3 s. A fixation cross was then presented on the screen. Participants then self-initiated the actual search image by fixating upon the cross and pressed a key. The cross hair image was not replaced by the search display until the cross hair was fixated and the key was pressed (i.e., gaze contingent stimulus presentation forcing central fixation at the start of the search image onset). Participants searched the image until they found the object, which they indicated by another key press. Participants were then instructed to continue to look at the object until the image was removed, which happened 3 s after object identification key press. A deadline of 30 s search time per trial was applied.

Search performance scores were based on pressing the button and fixating within the object boundary in the image. Specifically, if the fixation at key press was within a polygon 0.5 degrees larger than the target object boundary, the response was classified as a hit, otherwise it was a miss.

### Psychophysics Visual Acuity

To set an upper boundary on the functional field of view, we established the visual sensitivity for image features across the visual field. Specifically, we tested discrimination performance for all features used to create the stimuli images and calculated discrimination performance as a function of eccentricity (see Figure 8). Stimulus presentation was made contingent on center of image fixation using an eye tracker and a gaze contingent algorithm. The features were embedded in 27*20 grids similar to those used in the search task, with random features in all non target feature grid locations. A single participant (LH) made sequential two alternative forced choice recognition judgments on each feature from the library of features used to create the stimuli. A single trial consisted in the presentation of a fixation cross, followed by the presentation of the target feature. Then two stimulus images were presented with an intervening fixations cross. Finally, a spatial cue indicating the location at which to make the detection decision was presented. A total of 5520 trials were run (24 (fragment targets)×23(non targets)×5(eccentricities)×2(order)). Trials were organized in blocks by eccentricity, thus the location tested was known to the observer ahead of each trial even though the spatial cue was entered after the final comparison of each trial. As can be seen in figure 8, hit rate is virtually at chance at 8 degrees eccentricity hence an image patch of 17 degrees square corresponds to an upper bound on human identification performance.

### Object Detection Experimental Procedures

Eight university of Umeå students (age M = 25, five male) participated for monetary compensation of SEK 75 (about 10 USD). Testing was organized in three blocks, each consisting of a 40 trial detection test for a single object followed by 20 trials of object search trials, identical to the procedure used in the main experiment. Finally, the participants were again tested on a 40 trial detection test.

The detection test was designed as a sequential two alternative forced choice task (see figure 9). The test was organized in blocks of 40 detection trials. In the beginning of each block, the object to search for (i.e., the object cue) was presented intact in the center of the screen for 10000 ms. During each trial subjects viewed one image containing the cued object embedded in background features and another image containing background features only. Each image was presented for 300 ms. Participants responded by pressing keys after the final image in each pair indicating which image they thought contained the object (first or second). The images covered 27×20 degrees of participant’s visual field presented on an LCD monitor. Eye movements were recorded throughout (Eyelink 1000, 500 Hz). Three objects from the main search experiment were used (revolver, helmet and pear), one object in each test block. Each participant was tested on each of the three objects. Object block order was counterbalanced across participants. Images used in the detection tests appeared equally often in the first as the second test across participants. Each image (with and without embedded object) was constructed anew for each trial.

#### Image formation model

Each object constituted a cell grid *O*, forming a circuit of cells that fit within the image (see Figure 1A). Each object cell contained a feature *y*. The object template was randomly translated in the image grid, producing a set of features *y* at locations *x*, *{y, x+T}* with *T* being the offset from *x*. Each feature in y was then changed into a nonobject “background” feature with probability *K*. In the present study, *K* was fixed at.25. Background features were also placed at each cell in the image that was not in *O*. Background features were chosen as random samples from the empirical distribution of the object in the image. Formally, the probability of a feature in a certain cell in an image is given by:
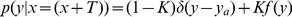
(1)


Where the probability *K* determines whether the feature is sampled from the background or the object template and.
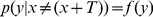
(2)for features outside of the object. *f(y)* is the empirical distribution of features, *δ* refers to the delta function and *y_a_* is the feature value of the object template at that cell.

#### Ideal observer

To test if practice made participants select more informative image regions, the information content of each image region must be established. This approach allows us to distinguish between information content in individual fixations as opposed to information acquired due to strategic selection. Other ideal observer models typically intermix those two features [4], [5].

We used an ideal observer model to assess the information available in individual eye fixations. The model only evaluates the information content at fixation, it does not select where to look next nor integrate information across fixations. In other words, the model only tells us how much can be learned about the object in a single fixation. Like humans, the model’s view was restricted to a window surrounding a simulated fixation point [5], [6]. We established an upper bound on the window size in a psychophysics pilot experiment (see psychophysics heading above). It showed that human observers receive no useful object fragment information beyond about eight degrees eccentricity. In the model, this field of view roughly corresponds to a 17×17 cell image patch. By design, the images in the task have no local information about the objects position and saliency is flat (i.e., an image patch of a single cell cannot tell object from background). Therefore, integration across several features is required to localize the object and correct integration requires object knowledge. Because visibility only allows a limited part of the image to be evaluated at once, an ideal model can only evaluate the image content by the fraction of the object template that fits within the model’s field of view. Therefore, the ideal observer’s object knowledge in this task was limited to all object portions that fit within the window. The model attempts to locate a cued fragmented object in the image by calculating the likelihood for the object portions at all possible locations and then picks the location and object portion with the highest likelihood as its response. We term a given portion of a chosen object, as viewed within the window, an object **subset** (Figure 10). The whole set of object subsets consists of all object portions that fit within the window.

For a square window *W* within the image, the model determines all features from object template locations *x_a_* that fits in the window. This is achieved by taking all non-empty intersections between the window *W* and the object template *O* as the template is translated over the image grid. Each set of features in the intersection then represents an object subset. We express all possible object subsets within a window as 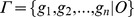.

The feature probabilities in a window are then determined by the subset that generated it i.e., for 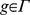
to have generated *W*. For window cells on the subset, the feature probabilities are again given by eq. 1 and 2. The probability that a certain subset *g* generated the features in the window is then given by:
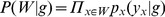
(3)


The probability that only background features produced the window content is given by:
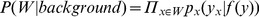
(4)


For each window location in the image, the model computed the probability that the object subset was present there and compared that to the probability that only background fragments were present. Formally, the model is described by the following equation to yield the likelihood ratio for the subset:
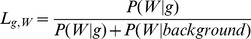
(5)


We let the highest likelihood over all subsets represent the likelihood of an object falling within that window size and location in an image, *ArgMax(L_Γ,w_).*

To determine if a window size is sufficient to locate the object, we test if the most likely subset over all subsets and window locations actually covered the original object in the image according to:
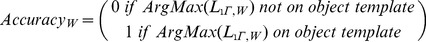
(6)


The results are displayed in Figure 4. The method allows for accuracy assessments of individual window locations, producing “1” for windows that locates the object and “0” otherwise. Naturally, many of these will have 0 accuracy, because the object did not intersect with the window. However, larger windows will at least have some small subset that intersects with the actual object location and therefore produces accurate responses (see also figure 10) to a higher extent than small windows.

The subset likelihood method allows for computing detection rate given a set of fixation locations and window size. The model’s best guess at object location is the image location that received the highest subset likelihood, across the set of fixations and all subsets. We use this likelihood measure as an indicator of object information present in the set of fixations. Note that although the likelihood will depend upon information as defined by information theory, it is not equivalent to this quantity.
